# Sensitivity analysis of slope stability based on eXtreme gradient boosting and SHapley Additive exPlanations: An exploratory study

**DOI:** 10.1016/j.heliyon.2024.e35871

**Published:** 2024-08-06

**Authors:** Hanjie Lin, Li Li, Yue Qiang, Yi Zhang, Siyu Liang, Xinlong Xu, Hongjian Li, Shengchao Hu

**Affiliations:** Department of Civil Engineering, Chongqing Three Gorges University, Wanzhou 404100, Chongqing, China

**Keywords:** Machine learning, XGBoost, SHAP, Sensitivity analysis of slope stability, A priori data-driven, GeoStudio

## Abstract

Slope instability through can cause catastrophic consequences, so slope stability analysis has been a key topic in the field of geotechnical engineering. Traditional analysis methods have shortcomings such as high operational difficulty and time-consuming, for this reason many researchers have carried out slope stability analysis based on AI. However, the current relevant studies only judged the importance of each factor and did not specifically quantify the correlation between factors and slope stability. For this purpose, this paper carried out a sensitivity analysis based on the XGBoost and SHAP. The sensitivity analysis results of SHAP were also validated using GeoStudio software. The selected influence factors included slope height (H), slope angle (β), unit weight (γ), cohesion (c), angle of internal friction (φ) and pore water pressure coefficient ($r_{u}$). The results showed that c and γ were the most and least important influential parameters, respectively. GeoStudio simulation results showed a negative correlation between γ, β, H, $r_{u}$ and slope stability, while a positive correlation between c, φ and slope stability. However, for real data, SHAP misjudged the correlation between γ and slope stability. Because current AI lacked common sense knowledge and, leading SHAP unable to effectively explain the real mechanism of slope instability. For this reason, this paper overcame this challenge based on the priori data-driven approach. The method provided more reliable and accurate interpretation of the results than a real sample, especially with limited or low-quality data. In addition, the results of this method showed that the critical values of c, φ, β, H, and $r_{u}$ in slope destabilization are 18 Kpa, 28°, 32°, 30 m, and 0.28, respectively. These results were closer to GeoStudio simulations than real samples.

## 1 Introduction

Slope engineering relates to mining, water conservancy, transportation and other projects, and slope stability is the core of slope engineering. In addition, slope failures can cause natural disasters such as collapses, landslides and debris flows [1,2]. Therefore, slope stability analysis has been a hot topic in the field of geotechnical engineering. Traditional slope stability analysis is mainly classified as limit equilibrium method [3], numerical analysis method [4,5] and limit analysis method [6]. However, the above 3 methods have limitations. Limit equilibrium methods are not sufficiently rigorous in theory and reliable in calculations due to their assumptions [7]. Numerical analysis methods are difficult to operate, making them difficult for engineers to master [8]. Limit analysis methods are difficult to determine the most dangerous damage mechanisms for homogeneous slopes [8]. In addition, the above methods need to be repeated in different study areas,and many parameters need to be set when using them [9,10]. Therefore, a faster, simpler and more accurate new approach for slope stability analysis is needed.

With the gradual development of AI, it provides new ideas for slope stability analysis. AI has the advantages of self-learning, nonlinear dynamic processing, evolutionary recognition and distributed representation [11]. In addition, it is a realistic, effective and intelligent new method of analysis [11]. Therefore, a large number of scholars have carried out research on slope stability prediction and factor sensitivity analysis based on AI. Table 1 demonstrated the main contributions of the above scholars.

**Table 1:** Important parameters of slope stability.

Authors	Main contributions
Khajehzadeh et al. [12]	They improved the artificial neural network to obtain a model that can be used to predict the safety factor of soil slopes under static and seismic loads.
Pham et al. [13]	They compared the performance difference between individual machine learning (ML) algorithms and ensemble algorithms in predicting slope stability. The results showed that the ensemble algorithm outperformed the individual ML algorithms. In addition, they strongly recommended to use extreme gradient boosting classifiers for slope stability analysis.
Karir et al. [14]	They used ensemble algorithms and individual machine learning algorithms to predict the safety factors of natural residual soil slopes and anthropogenic overburden mine dump slopes. The results similarly showed that ensemble algorithms outperform individual ML algorithms. In addition, they indicated that in the case of anthropogenic landfill slopes, the efficiency of various machine learning models in predicting the safety factor is better than natural residual soil slopes.
Wang et al. [15]	They proposed an improved support vector machine model for slope stability prediction by optimizing the penalty parameter C and kernel function parameter.
Mahmoodzadeh et al. [16]	They used 6 machine learning algorithms to predict the safety factor of slopes. The results showed that the Gaussian process regression possessed a higher goodness of fit and lower error than the remaining 5 models. Furthermore, they indicated that the angle of internal friction and unit weight were the most and least effective parameters for slope stability, respectively.
Lin et al. [17]	They compared the ability of 11 ML algorithms to predict slope safety factors. The results showed that the prediction performance of the nonlinear regression method was slightly better than the linear regression method. In addition, they indicated that cohesion and slope height were key factors affecting slope stability.
Nanehkaran et al. [18]	They compared the prediction performance of the Multi-layer Perceptron (MLP), Decision Tree (DT), Support Vector Machine (SVM), and Random Forest (RF) algorithms on slope safety coefficients. The results showed that MLP possessed high accuracy and precision.

The analysis in Table 1 showed that various AI algorithms exhibited excellent potential for predicting slope stability. Moreover, the prediction results of the ensemble algorithms were better than the individual ML algorithms. This is because ensemble learning enhances the performance of ML models by accepting the diversity of their underlying predictor variables to learn different perspectives of the database. In addition, there are fewer current studies on slope stability sensitivity analysis based on machine learning algorithms. And the conclusions of these studies lack validation. For example, Lin et al. and Mahmoodzadeh et al. disagreed on the discrimination of the main factors, as different datasets also lead to different rankings of factor importance. In addition, the existing studies have only judged the importance of influencing factors but did not specifically quantify the correlation between influencing factors and slope stability.

The emergence of the Ex-post Explanation Method (EEM) provides a new solution to the above shortcomings. This approach works by continually making assumptions and testing to infer the working principles of models. The EEM comprises Local Interpretable Model-agnostic Explanation (LIME) and the SHapley Additive exPlanations (SHAP), etc. However, SHAP exhibits better sampling stability and variability than LIME [19,20]. Therefore, SHAP was widely used in other fields. For example, Parsa et al. [21] constructed an interpretable model for traffic accidents based on eXtreme Gradient Boosting (XGBoost) and SHAP. The results showed that SHAP determined the relationship between the influencing factors and the probability of traffic accidents. Yang et al. [22] explored the relationship between built environment factors and the spatial distribution of truck-related crashes based on XGBoost and SHAP. The results showed that the importance of the influencing factors presented variability in different localizations. Jas et al. [23] developed a model for liquefaction potential assessment of soil based on XGBoost and SHAP. The results showed that interpretable algorithms can bridge the gap between liquefied traditional domain knowledge and soft computing methods.

In summary, as an ensemble learning algorithm, XGBoost showed high accuracy, generalization ability, and fast operation speed compared to complex individual machine learning algorithms [24]. Therefore, the ensemble algorithm has been widely used in the field of geotechnical engineering [[25], [26], [27]]. In addition, SHAP was able to explain the relationships captured by machine learning models through relative importance and partial dependence maps. But unfortunately, there are almost no studies using SHAP for sensitivity analysis of slope stability. Moreover, it is not clear whether the results of SHAP are consistent with the results of traditional computational methods. Therefore, the method has not yet been fully recognized in the field relevant to this study.

For this purpose, this paper constructed an interpretable prediction model for slope stability based on XGBoost and SHAP. The Optuna, Particle Swarm Optimization (PSO) and Whale Optimization Algorithm (WOA) were used to optimize the key hyperparameters of XGBoost. Then, SHAP was used to determine the importance ranking of factors and the dependencies between factors and slope stability. The selected influence factors included slope height, slope angle, unit weight, cohesion, angle of internal friction and pore water pressure coefficient. Finally, the above results of SHAP were verified by comparing with the results of GeoStudio numerical simulation software. The main contributions of this paper were as follows.

(1) The prediction results of Optuna-XGBoost, PSO-XGBoost and WOA-XGBoost were compared, which can provide some references for slope stability prediction.
(2) A comparison with conventional numerical simulation software revealed a potentially misleading dependence between factors and slope stability in the real sample case.
(3) This paper provided potential solutions to the above possible misleading.

In Section 2, the data, the used methods, the evaluation metrics, and the implementation steps of the GeoStudio numerical simulation were presented. In Section 3, this paper presented the predictions of the Optuna-XGBoost, PSO-XGBoost and WOA-XGBoost models, the results of SHAP sensitivity analysis and the results of the numerical simulation software. In Section 4, this paper discussed the prediction performance of models, sensitivity analysis results, potential solutions and limitations. In Section 5, the main conclusions of this paper were presented.

## 2 Data and methods

### 2.1 Data

#### 2.1.1 2.2.1 input parameter selection

Slope stability is affected by topography, geological structure, lithology, rainfall, earthquake and weathering. This effect can generally be divided into 3 aspects: the geotechnical parameters of the rock and soil, the slope geometry and the water action in the rock and soil [28]. Numerous researchers [[29], [30], [31]] have chosen slope height (H), slope angle (β), unit weight (γ), cohesion (c) and angle of internal friction (φ) as typical slope parameters. However, they did not consider the effect of pore water pressure coefficient ($r_{u}$). The $r_{u}$ directly affects the strength and susceptibility of the soil to liquefaction, which further affects the stability of the slope [32]. Therefore, this paper selected H, β, γ, c, φ and $r_{u}$ as the typical parameters affecting slope stability, and each parameter definition is shown in Table 2.

**Table 2:** Important parameters of slope stability.

Factors	Definition
H	The vertical distance between the slope base to the slope crest
β	The angle between the inclined slope plane and the slope base plane
$r_{u}$	The coefficient of the relationship between pore water pressure increments and stress increment under undrained condition
γ	The weight per unit volume of the soil/rock
c	The part of shear strength that is irrelevant to the normal effective stress during soil/rock movements
φ	The dip angle of shear strength line in σ-τ coordinate plane

#### 2.1.2 Data preprocessing

**Fig. 1:**
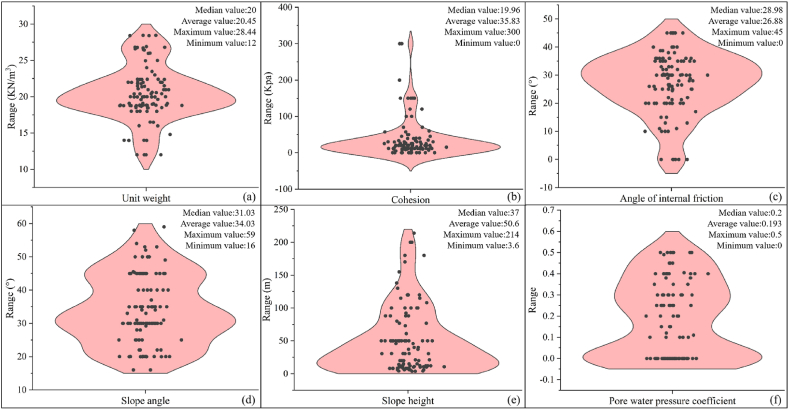
Statistical map of the data (a: Unit weight; b: Cohesion; c: Angle of internal friction; d: Slope angle; e: Slope height; f: Pore water pressure coefficient).

A total of 129 sets of slope data were collected in this paper, of which 107 sets were from the literature [[33], [34], [35]], as shown in Appendix A. These 107 sets of data were used as training and validation sets, and the remaining 22 sets of data were used as test sets. The percentage of training set, test set and prediction set were 80 %, 10 % and 10 % respectively. The data statistics were shown in Fig. 1, including the distribution of factors, minimum, maximum, median and average values.

The Pearson Correlation Coefficient (PCC) is a metric utilized in statistics to measure the strength and direction of the linear relationship between two variables. It has a value between −1 and 1: 1 indicates a perfect positive correlation; −1 indicates a perfect negative correlation; and 0 indicates no linear correlation. This method can be used for multiple covariance detection by observing whether there is a strong linear relationship between the input factors. In addition, the PCC can improve the stability and interpretive reliability of models avoiding misleading results. The formula of PCC was shown as follows:

(1) $$PCC=\frac{\sum \left(X_{i}-\bar{X}\right)\left(Y_{i}-\bar{Y}\right)}{\sqrt{\sum {\left(X_{i}-\bar{X}\right)}^{2}\sum {\left(\left(Y_{i}-\bar{Y}\right)\right)}^{2}}}$$

**Fig. 2:**
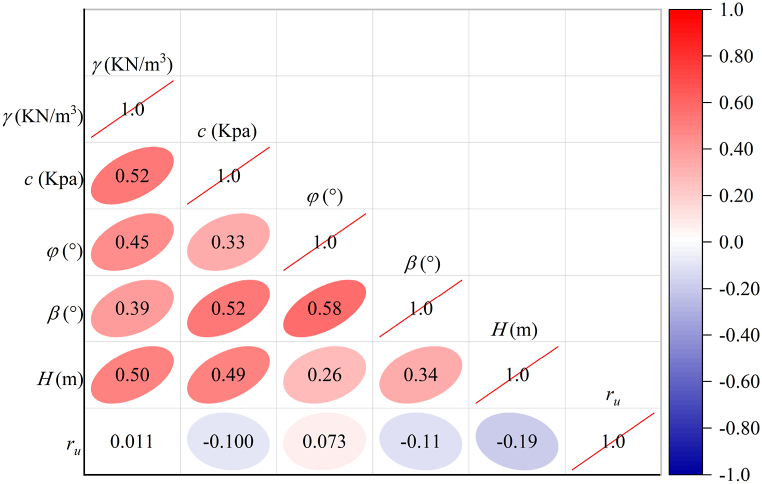
PCC results for each input factor.

Where $X_{i}$ and $Y_{i}$ are the observed values of the 2 factors; $\bar{X}$ and $\bar{Y}$ are the average values of the 2 factors. The PCC results for each input factor were shown in Fig. 2. The results showed that the PCC of internal friction angle with slope angle was the largest with 0.58, and the PPC of unit weight with pore water pressure coefficient was the smallest with 0.011. Therefore, there was no significant covariance between the factors.

Finally, data cleansing was used on all data, including processing of missing values and handling of repeating data. This can increase data availability, reduce redundancy and bias, and enhance model performance. The results showed that all data had no missing values, but data No.74 and No.75 were duplicates.

### 2.2 Methods

#### 2.2.1 XGBoost model

The XGBoost algorithm is based on the concept of gradient boosting decision making and was proposed by Chen and Guestrin [36] in 2016. The XGBoost algorithm performs well when dealing with dependent variable classification problems [37]. In addition, the boost tree of XGBoost may exceed its computational limits. This is because this algorithm enhances the process of loss function and loss optimization in ML. Therefore, the XGBoost algorithm has the advantages of high operational efficiency and accuracy [36]. In this algorithm, the training of the boosting tree is based on the residual. The prediction of n-1 is used as input for n, which is the residual [38]. The goal of each training is to make the residuals smaller and smaller [38]. In addition, the XGBoost is different from traditional ensemble decision tree algorithms. Because the regular term in its loss function not only controls the complexity of the model, but also prevents the model from overfitting [39]. The objective function for slope stability prediction is shown in Formula (2) [24].

(2) $$Obj=-\frac{1}{2}\sum_{T}^{j=1}\frac{G_{j}^{2}}{H_{j}+\lambda }+\gamma T$$

Where λ represents the fixed coefficient; γ represents the complexity parameter; T represents the number of leaf nodes in the tree; $G_{j}$ represents the sum of the first-order partial derivatives of the samples contained in leaf node j; $H_{j}$ denotes the sum of the second order partial derivatives of the sample contained in the leaf node j [24].

#### 2.2.2 Hyperparameter optimization

Hyperparameters are the frame parameters of the ML model. Suitable hyperparameters can improve the effectiveness and performance of learning to obtain the optimally performing model on a given dataset. The key hyperparameters of XGBoost included *n_estimators*, *learning_rate*, *max_depth*, and *gamma*. Where *n_estimators* is the number of trees in the decision tree, also known as the number of base evaluators. This parameter is related to the prediction effectiveness of models, and training time. The *learning_rate* is the learning rate of models, which represents the magnitude size of each parameter update. This parameter controls the convergence rate and runtime of models. The *max_depth* is the maximum depth of the decision tree, representing the length of the longest path that the decision tree can generate. This parameter controls the underfitting or overfitting effectiveness of models. The gamma is the decreasing value of the minimum function required for node splitting. This parameter controls the fit and complexity of models.

The Optuna is an automatic hyperparameter adjustment framework, which includes grid search method, stochastic search method, Bayesian optimization algorithms, etc [40]. The Bayesian optimization method has proved to be an effective optimization algorithm and was widely used in hyperparametric optimization [41]. In addition, the metaheuristic algorithms were also often used to optimize hyperparameters, due to the strong global search capability [42,43]. Particle Swarm Algorithm (PSO) [44] was an optimization algorithm based on group intelligence, which simulated the behavior of the birds foraging for food. The basic idea of the PSO was to find the optimal solution by moving individuals (called particles) through the solution space. The Whale Optimization Algorithm (WOA) [45] was an optimization algorithm based on the hunting behavior of whales, which simulated the process of humpback whales hunting through “bubble nets”. The WOA consisted of three main operations: circling the prey, spiraling to update the position, and searching for the prey. The number of whales and the number of iterations in the WOA of this paper were 30 and 50, respectively. And the number of particles, number of iterations, inertia weight, individual learning factor and social learning factor in PSO were 30, 50, 0.5, 1.5, 1.5 respectively.

In summary, the key hyperparameters of XGBoost were optimized by using Optuna, PSO and WOA, as shown in Table 3.

**Table 3:** The hyper-parameters of XGBoost.

Optimization algorithm	Hyper-parameter Search space	Hyperparameter results
Optuna	*n_estimators*: (100, 2000) *learning_rate*: (0.01, 1.0) *max_depth*: (1, 10) *gamma*: (0.01, 1.0)	*n_estimators*:1066; *learning_rate*: 0.4078011379179168; *max_depth*: 2; *gamma*: 0.252543073674663.
PSO		*n_estimators*: 797; *learning_rate*: 0.0735994687503164; *max_depth*: 3; *gamma*: 0.04395047730601182.
WOA		*n_estimators*: 150; *learning_rate*: 0.015094630312803206; *max_depth*: 1; *gamma*: 0.015094630312803206.

#### 2.2.3 Evaluation indicators

In this study, 10 fold cross validation was chosen as the evaluation metric for the validation sets. In addition, confusion matrix, Receiver Operating Characteristic (ROC), Area Under Curve (AUC), Cohen's Kappa, Accuracy, F-score, True Positive Rate (TPR), False Positive Rate (FPR), and running time were simultaneously used as evaluation criteria for the test sets.

**Fig. 3:**
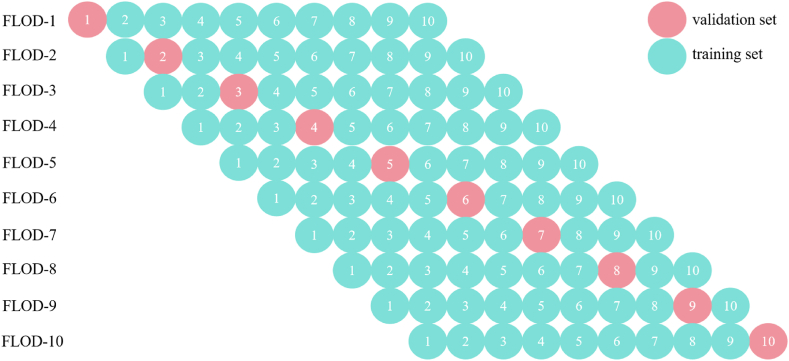
Schematic of the 10 fold cross validation.

The 10 fold cross validation is a statistical method of cutting data samples into 10 subsets, commonly used to evaluate the performance of machine learning models. In 10 fold cross validation, 9 subsets were usually used to train models while the remaining 1 subset was used to test models. This process was repeated 10 times, with each subset getting a chance to be used as a validation set. Then, the results of an average of 10 experiments can be calculated to obtain the final model performance. This approach helps us to more accurately understand the performance of models on unknown data. The 10 fold cross validation was shown in Fig. 3.

The confusion matrix is a fundamental tool for evaluating the performance of classification models, especially for binary and multiclassification problems. It showed the relationship between the predictions of models and the actual results in the form of a matrix, as shown in Table 4. The *TP* was the number of samples that were correctly predicted as positive classes by models. The *FP* was the number of samples that were incorrectly predicted as positive classes by models, but were actually negative classes. The *FN* was the number of samples that were incorrectly predicted as negative classes by models, but were actually positive classes. The TN was the number of samples correctly predicted as negative classes by models.

**Table 4:** Confusion matrix.

real value	predicted value	
	Negative(N)	Positive(P)
Negative(N)	True Negative (TN)	False Positive (FP)
Positive(P)	False Negative (FN)	True Positive (TP)

**Table 5:** Values of factors used for GeoStudio validation.

	γ (KN/m3)	c (Kpa)	φ (°)	β (°)	H (m)	$r_{u}$
Maximum value	28.44	300	45	59	214	0.5
Minimum value	12	0	0	16	3.6	0
Median value	20	19.96	28.98	31.01	37	0.2
Change size	1.644	30	4.5	4.3	21.04	0.05

The *TPR*, also known as Sensitivity or Recall, was a measure of the ability of models to correctly identify positive class samples. And *FPR*, also known as 1-Specificity, is a measure of the ability of models to misidentify negative class samples. The formulas of *TPR* and *FPR* were shown as follows:

(3) $$TPR=\frac{TP}{TP+FN}$$

(4) $$FPR=\frac{FP}{FP+TN}$$

The ROC curve was an important tool for evaluating the performance of the binary classification model. It demonstrated the performance of models under different thresholds, helping us to understand the advantages and disadvantages of models under different classification criteria. The ROC curve took FPR as the horizontal axis, and TPR as the vertical axis. The AUC was the value of the area under the ROC curve, commonly used to measure the overall performance of classification models. The range of values for AUC was 0–1, with larger values indicating better model performance.

The *Accuracy* was the proportion of the number of samples correctly predicted by models to the total number of samples. It was an intuitive metric to measure the overall predictive power of models. However, in the case of category imbalance, the accuracy may be misleading. The *Accuracy* was shown in the following formula:

(5) $$Accuracy=\frac{TP+TN}{TP+TN+FP+FN}$$

The *F*1 *score* was the tempered average of precision and recall. It combined the precision and recall capabilities of models with particular applicability to the case of category imbalance. In the case of category imbalance, the *F*1 *score* was better reflecting the true performance of models than the *accuracy*. The *F*1 *score* was shown in the following formula:

(6) $$F1\;Score=\frac{2TP}{2TP+FP+FN}$$

The *Cohen's Kappa* was a statistical method used to measure the consistency or reliability between two observers. It considered random consistency, which was more accurate than simple percentage consistency. The *Kappa* value ranged from −1 to 1, where 1 indicated perfect consistency, 0 indicated random consistency, and a negative value indicated a consistency below the random level. Using it to evaluate binary classification models can better reflect the performance of models, especially in the case of category imbalance. The *Cohen's Kappa* was shown in the following formula:

(7) $${Cohen}^{\prime }s\;Kappa=\frac{P_{o}-P_{e}}{1-P_{e}}$$

Where $P_{o}$ was the calculated observational consistency, which was the proportion of model predictions that agreed with the actual results. The calculation process for $P_{o}$ was the same as for *Accuracy*. The $P_{e}$ was calculated expected consistency, which was the expected consistency based on the probability of the randomized prediction. The $P_{e}$ was shown in the following formula:

(8) $$P_{e}=\left(\frac{\left(TP+FP\right)∙\left(TP+FN\right)}{{\left(TP+TN+FP+FN\right)}^{2}}\right)+\left(\frac{\left(TN+FN\right)∙\left(TN+FP\right)}{{\left(TP+TN+FP+FN\right)}^{2}}\right)$$

#### 2.2.4 Shapley additive exPlanations (SHAP)

XGBoost is a complex ensemble decision tree model, and its prediction process is difficult to understand intuitively. Therefore, these complex algorithms suffer from poor interpretability. This problem is also known as the “black-box model”, which is the main reason for hindering the application of machine learning algorithms in engineering practice [24]. Therefore, it is necessary to interpret the results of the prediction. The SHAP interpretable algorithm can solve the above problem, which was proposed by Lundberg and Lee in 2017 [19]. The core concept of SHAP was to calculate the marginal contribution of features to the output values. This method took all features as contributors and calculates their contribution values. Subsequently, the sum of contribution values from all features was the final prediction, which was shown in formula (9) [19]. This method can interpret “black-box models” in local and global aspects, thus interpreting the prediction results of ML algorithms more efficiently and reliably.

(9) $$f\left(x\right)=g\left(z^{*}\right)=\varphi_{0}+\sum_{i=1}^{M}\varphi_{i}z_{i}^{*}$$

In the above formula, f(x) is the machine learning model, in this study it is XGBoost; $z^{*}$ = {0,1}, when the feature is observed, $z^{*}$ = 1, otherwise it = 0; If i is involved in the prediction process, M is the number of features; $\varphi_{0}$ is the base value; $\varphi_{i}$ is the contribution of feature i. The expression for $\varphi_{i}$ was shown in formula (10) [46].

(10) $$\varphi_{i}=\sum_{S\subseteq N\backslash i}\frac{\left|S\right|!\left(M-\left|S\right|-1\right)!}{M!}\left[f\left(S\cup \left\{i\right\}\right)-f\left(S\right)\right]$$

In the above formula, N is the set of all input features; S is the set containing the non-zero index in $z^{*}$; f(S∪{i}) and f(S) are model results with or without the first feature, respectively [46].

#### 2.2.5 GeoStudio for validation

**Fig. 4:**
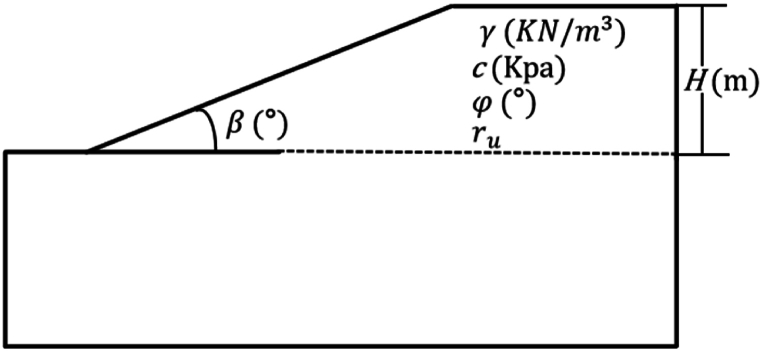
GeoStudio slope model.

In this paper, the results of SHAP were validated with reference to the method of Kostić et al. [47]. The numerical simulation software was the GeoStudio 2018 R2, the analysis type of the software was Morgenstern-Price, the interstrip force function used half-sine, and the pore water pressure used $r_{u}$ coefficients, the GeoStudio slope model was shown in Fig. 4. The specific steps were as follows:

(1) The statistics of maximum, minimum and median values of each impact factor were presented.
(2) One factor was selected, and the other factors were set to median values. Controlling the gradual change of the selected factor, the remaining factors were kept constant. The gradual change size = (Maximumvalue−Minimumvalue)/10.
(3) The range of variation of the slope safety factor was used to determine the importance of the influencing factors. The specific values of the above factors were shown in Table 5.

**Fig. 5:**
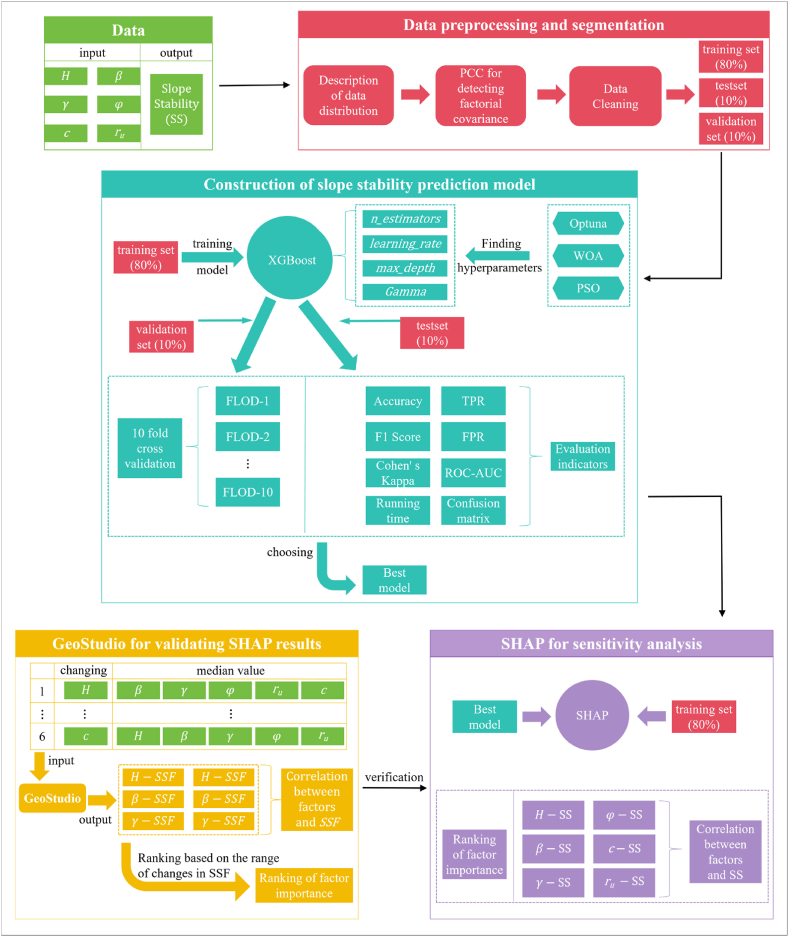
Workflow diagrams.

Firstly, this paper selected H, β, γ, c, φ and $r_{u}$ as the input parameters of slope stability (SS) based on 129 slope data sets. Subsequently, the distribution of the data was described, the data was subjected to multicollinearity detection and data cleaning, and the data was divided into the training sets, test sets, and validation sets. Later, the key hyperparameters of XGBoost were optimized by using Optuna, PSO and WOA, 10-fold cross-validation was used for the validation sets, and 8 metrics were used for the comprehensive evaluation of the test sets. The optimal model was selected based on the evaluation results of the test and validation sets. The training set was interpreted by using SHAP based on the optimal model for judging the factor importance and the relationship between each factor and SS. Finally, the results of SHAP were validated by using GeoStudio software simulation to generate slope safety factor (SSF). The entire workflow of this paper was shown in Fig. 5.

## 3 Results

### 3.1 Model prediction results

The 10-fold cross-validation results for the validation sets were shown in Table 6. The results showed that the average accuracies of the 10-fold cross-validation for the validation sets of Optuna-XGBoost, WOA-XGBoost, and PSO-XGBoost were 0,9136, 0.6806, and 0.9056, respectively. In the validation set, Optuna-XGBoost exhibited better accuracy than the other two models.

**Table 6:** 10-fold cross validation results for the testing set.

	Average value		
	PSO-XGBoost	WOA-XGBoost	Optuna-XGBoost
Flod-1	0.8889	0.6667	0.8000
Flod-2	1.0000	0.6667	0.8182
Flod-3	0.8889	0.5556	1.0000
Flod-4	0.8889	0.8889	0.9091
Flod-5	0.8889	0.7778	1.0000
Flod-6	0.8750	0.6250	0.9091
Flod-7	1.0000	0.3750	0.8000
Flod-8	1.0000	0.7500	1.0000
Flod-9	0.8750	0.7500	1.0000
Flod-10	0.7500	0.7500	0.9000
Average value	0.9056	0.6806	0.9136

**Fig. 6:**
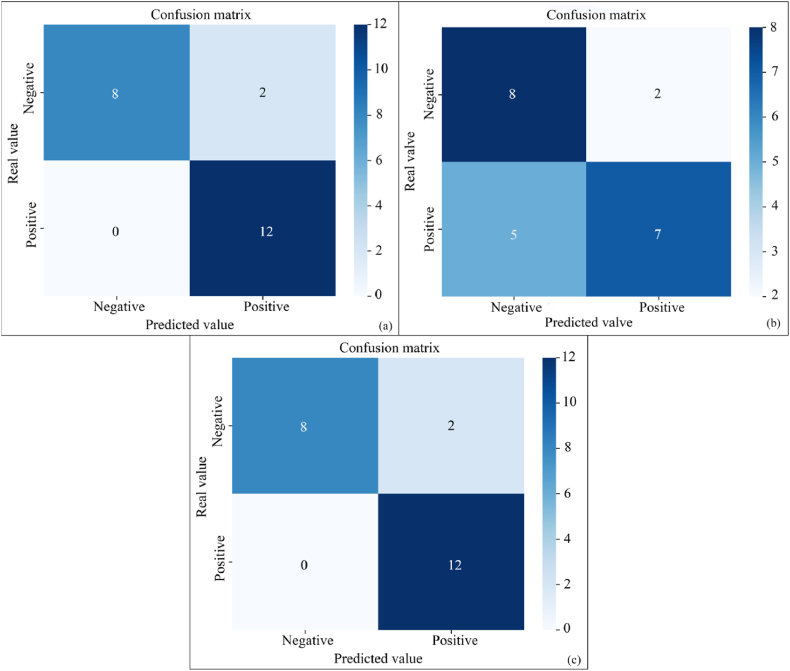
Confusion matrix results (a: Optuna-XGBoost; b: WOA-XGBoost; c: PSO-XGBoost).

The confusion matrix was used to show the prediction effectiveness of Optuna-XGBoost, WOA-XGBoost and PSO-XGBoost for the test set data, as shown in Fig. 6. The results showed that Optuna-XGBoost and PSO-XGBoost correctly judged 20 groups of slopes, but judged 2 groups of stable slopes as unstable. The WOA-XGBoost correctly judged 15 groups of slopes, but judged 5 groups of stable slopes as unstable, as well as 2 groups of unstable slopes as stable.

**Fig. 7:**
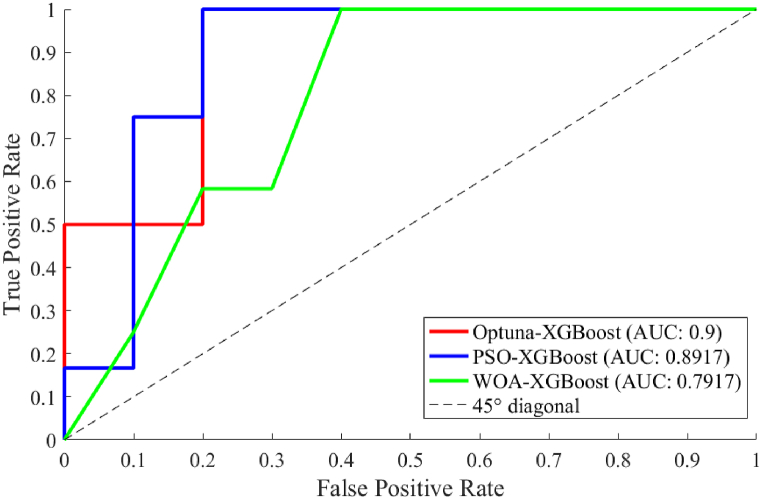
ROC-AUC results.

The prediction results of Optuna-XGBoost, WOA-XGBoost and PSO-XGBoost were plotted as ROC-AUC curves, as shown in Fig. 7. The results showed that Optuna-XGBoost, WOA-XGBoost and PSO-XGBoost had AUC values of 0.9, 0.8917, and 0.7917, respectively. In addition, the ROC curves of Optuna-XGBoost and PSO-XGBoost were closer to the upper left corner than the WOA-XGBoost.

**Fig. 8:**
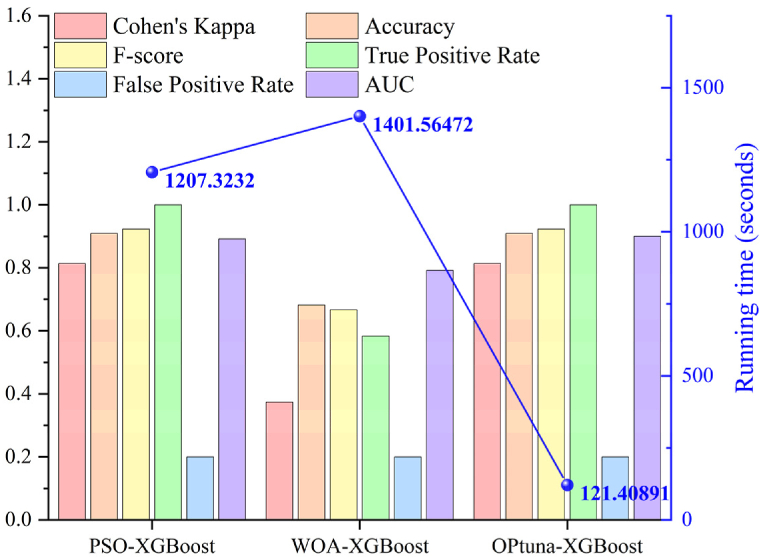
Evaluation metrics results for test sets of Optuna-XGBoost, WOA-XGBoost and PSO-XGBoost.

The AUC, Cohen's Kappa, Accuracy, F-score, TPR, FPR, and running time for the test sets of Optuna-XGBoost, WOA-XGBoost, and PSO-XGBoost were shown in Fig. 8. The Optuna-XGBoost and PSO-XGBoost had the same Cohen's Kappa, Accuracy, F-score, TPR and FPR values, which were 0.8136, 0.9091, 0.9231, 1 and 0.2, respectively. However, the AUC value and running efficiency of PSO-XGBoost were inferior to Optuna-XGBoost. The AUC value and running time of Optuna-XGBoost were 0.9 and 121.40891 s, while the AUC value and running time of PSO-XGBoost were 0.8917 and 1207.3232 s, respectively. In addition, the AUC, Cohen's Kappa, Accuracy, F-score, TPR, FPR, and running time of WOA-XGBoost were 0.7917, 0.374, 0.6818, 0.6667, 0.5833, 0.2, and 1401.56472 s, respectively.

### 3.2 Sensitivity analysis results

**Fig. 9:**
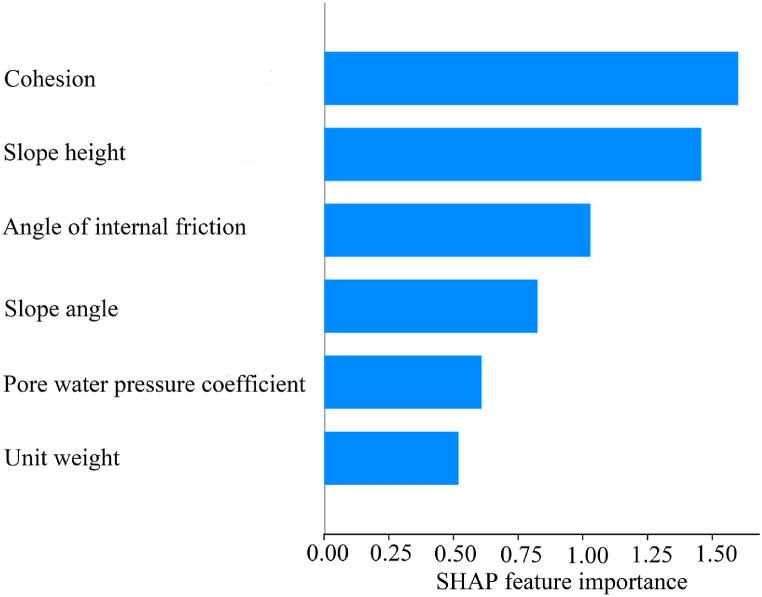
Ranking of importance determined by SHAP.

Based on the training set and the optimal model, the importance ranking was performed by calculating the average of the SHAP values for each influencing factor, as shown in Fig. 9. The left side, from top to bottom, was the importance of the feature, and the horizontal axis was the result of averaging the absolute values of the SHAP values for each sample. The results showed the importance of the factors in the following order: cohesion > slope height > angle of internal friction > slope angle > pore water pressure coefficient > unit weight.

**Fig. 10:**
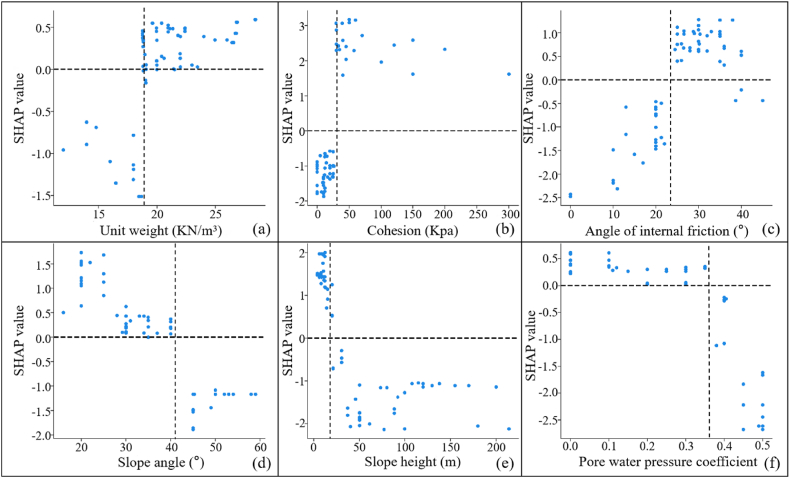
Single factor analysis determined by SHAP (a: Unit weight; b: Cohesion; c: Angle of internal friction; d: Slope angle; e: Slope height; f: Pore water pressure coefficient).

The positive and negative relationship between each influencing factor and slope stability were reflected by Fig. 10. In Fig. 10, the horizontal axis was the actual value of the factor itself, and the vertical axis was the SHAP value of the corresponding output. When the SHAP value was greater than 0, it indicated that the factor values had a positive effect on the prediction results. Taking Fig. 10(a) as an example, the SHAP value rose with increasing unit weight, and when unit weight = 18 kN/m^3^, the SHAP value = 0. This showed that the unit weight was positively correlated with the slope stability prediction, and that the slope was in a critical state when the unit weight = 18 kN/m^3^. Similarly, the cohesion and angle of internal friction were also positively correlated with the slope stability prediction, with critical values of 30 Kpa and 24°, respectively. While the Slope angle, slope height and pore water pressure coefficient were negatively correlated with the slope stability prediction, the critical values were 42°, 20m and 0.37, respectively.

**Fig. 11:**
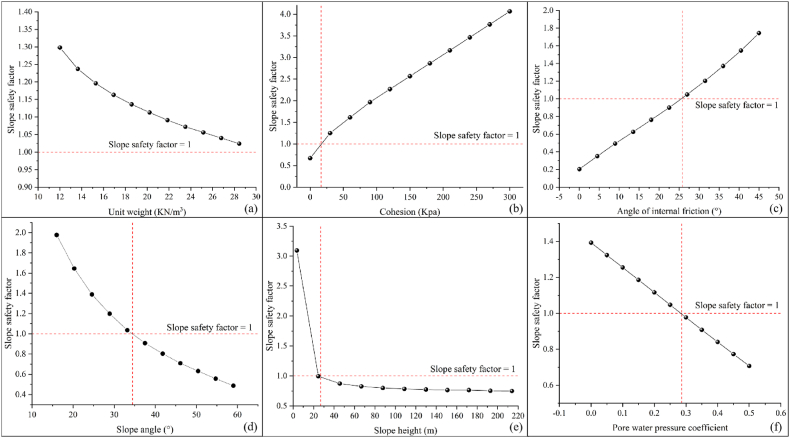
Single factor analysis determined by GeoStudio (a: Unit weight; b: Cohesion; c: Angle of internal friction; d: Slope angle; e: Slope height; f: Pore water pressure coefficient).

The slope safety factor was simulated by using GeoStudio software to determine the relationship between each factor and slope stability, as shown in Fig. 11. The results showed that the unit weight, slope angle, slope height and pore water pressure coefficient were negatively correlated with slope stability, while cohesion and angle of internal friction were positively correlated with slope stability. In addition, the critical values of cohesion, angle of internal friction, slope angle, slope height, and pore water pressure coefficient were 20 Kpa, 26°, 34°, 28 m, and 0.29, respectively.

**Fig. 12:**
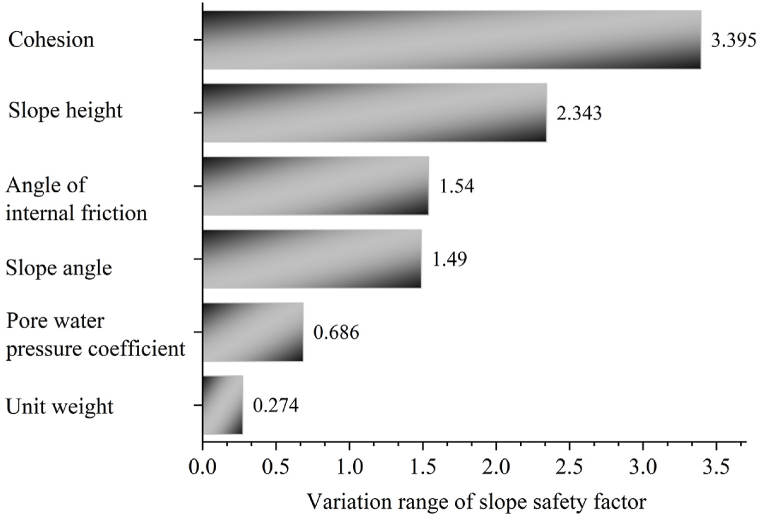
Ranking of importance determined by GeoStudio.

The importance of each factor was determined based on the variation range of the slope safety factor for each factor in Fig. 11, as shown in Fig. 12. The results showed the importance of the factors in the following order: cohesion > slope height > angle of internal friction > slope angle > pore water pressure coefficient > unit weight.

## 4 Discussions

The 10 fold cross validation can effectively reduce the risk of model overfitting to specific training data, providing a more stable and reliable result for model performance evaluation, especially when the data volume was limited. The results showed that Optuna-XGBoost had better overfitting resistance, more stable and reliable prediction performance than WOA-XGBoost and PSO-XGBoost.

In the test sets, this paper used AUC, Cohen's Kappa, Accuracy, F1-score, TPR, FPR, and running time to comprehensively evaluate the performance of models. The results showed that Optuna-XGBoost and PSO-XGBoost had better prediction effects compared to WOA-XGBoost. However, the running efficiency of the PSO-XGBoost was inferior compared to the Optuna-XGBoost, and its running time was 9.94 times longer than the Optuna-XGBoost. Similarly, the running time of the WOA-XGBoost was 11.54 times longer than the Optuna-XGBoost.

All of the above results indicated that the Optuna-XGBoost had better prediction performance and resistance to overfitting, occupied fewer computational resources, had faster operational response, and provided better scalability on large-scale datasets.

The Optuna supported parallelized searches that can be run on multiple machines or multiple threads simultaneously to speed up the optimization process. And this method made the hyperparameter tuning process more efficient and easier to use. In contrast, PSO and WOA were group intelligence algorithms that relied on the collaborative search of multiple individuals (particles or whales) to find an optimal solution. Each individual was updated iteratively in the search space, gradually approximating the optimal solution. This is effective but may require a large number thereof iterations and individuals to find a satisfying solution in a high dimensional and complex search space, resulting in longer running times. In addition, Optuna boasted an early termination mechanism compared to PSO and WOA, which can save a lot of computational resources and time.

The results in Fig. 9, Fig. 12 were compared to show that the ranking of importance of factors was: cohesion > slope height > angle of internal friction > slope angle > pore water pressure coefficient > unit weight. However, it is worth noting that the values of the angle of internal friction and slope angle in Fig. 12 exhibited slight differences from Fig. 9. The reasons for this discrepancy can be divided into two main aspects. On the one hand, the benchmark model for SHAP in this paper was a classification model, whereas GeoStudio calculated the slope safety factors. The calculated slope safety factors were more oriented towards continuous variables, which can be regarded as a regression model. On the other hand, the data explained by SHAP was the training sets, while GeoStudio calculated the slope safety coefficients of all the sample data. But importantly, the SHAP correctly judged the importance of factors, which was consistent with the ranking results of GeoStudio.

Comparing the results of Fig. 10, Fig. 11 were shown in Table 7. The results showed that the unit weight had a large difference in the results. The SHAP determined that there is a positive correlation between the unit weight and the slope stability, while GeoStudio determined that there is a negative correlation between the unit weight and the slope stability. The theoretical analysis revealed that the increase of unit weight will lead to the increase of the sliding force of soil body, which will lead to the decrease of slope stability. Therefore, the correlation between unit weight and slope stability determined by GeoStudio was more in line with the real situation. But surprisingly, the results of SHAP and GeoStudio were consistent regarding the correlation of other factors with slope stability. Cohesion is the mutual attraction between soil particles, which can help to increase the shear strength of soil, thus improving the slope stability. The angle of internal friction is the friction between soil particles, which reflects the ability of soil to resist shear deformation. A high shear strength indicates that the slope is able to withstand greater shear stresses, thus improving the slope stability. A larger slope angle not only means higher shear stresses in the slope, but also makes the gravity component more inclined to work in the direction of slopes. Both of these reasons can cause the slope stability to deteriorate. An increase in slope height means an increase in the weight of soil in the slope, which can potentially increase the likelihood of sliding. In addition, higher slopes can produce greater shear stresses, especially at the bottom of slopes. This will increase the risk of the soil sliding along the sliding surface, thus reducing the stability of the slope. And an increase in pore water pressure coefficient can reduce the effective stress of soil, thus reducing the shear strength of soil and increasing the risk of soil sliding. The above theoretical analysis showed that the results of GeoStudio simulation were more in line with the actual situation, while SHAP misjudged the correlation between unit weight and slope stability.

**Table 7:** Comparative results of Fig. 11, Fig. 12.

Factors	The correlation between factors and slope stability	
	SHAP results	GeoStudio results
Unit weight	positive correlation	negative correlation
Cohesion	positive correlation	positive correlation
Angle of internal friction	positive correlation	positive correlation
Slope angle	negative correlation	negative correlation
Slope height	negative correlation	negative correlation
Pore water pressure coefficient	negative correlation	negative correlation

**Fig. 13:**
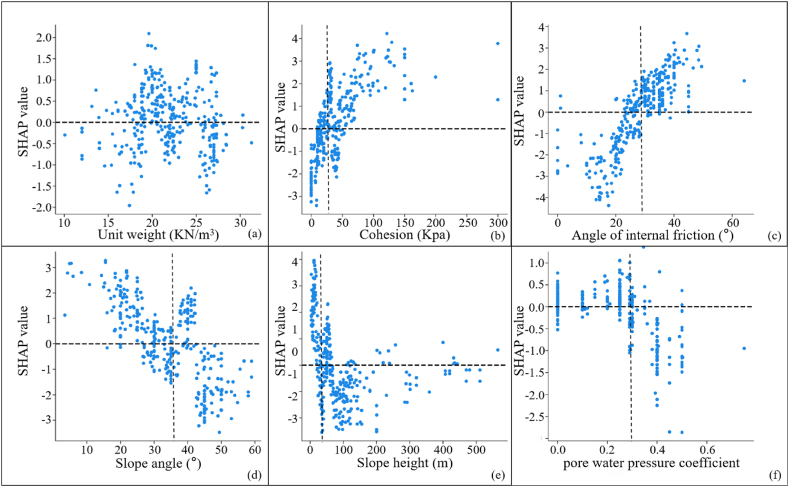
Single factor analysis of 449 groups of data determined by SHAP (a: Unit weight; b: Cohesion; c: Angle of internal friction; d: Slope angle; e: Slope height; f: Pore water pressure coefficient).

The calculation of SHAP values depends on the predictions of models and the distribution of input data. And the limited sample may not adequately represent the distribution of the real data, thus affecting the accuracy of SHAP values. For this reason, the sample data in this paper was expanded to 449 groups of slope data as shown in Appendix B. The 449 groups of data were brought into the model of this paper to recalculate the SHAP values, and the results were shown in Fig. 13. The results showed that there was no significant correlation between unit weight and slope stability, and the data were aggregated throughout. This may be due to the fact that the sample data contained more noise and outliers, leading to an increase in the volatility of the SHAP values, thus reducing their interpretability.

In addition, some dirty data mixed with real samples may make SHAP unable to capture valid information. Whereas the results of the SHAP model depended on the predictions of the model and the distribution of the input data. Thus, dirty data can change the true logic between input and output, thereby causing the correlation between unit weight and slope stability to defy common sense logic. Another reason could be that current AI cannot effectively (and causally) infer the possible consequences of actions without the relevant priori data of stable predictive pattern [48].

For this reason, this paper used the priori data driven approach to improve the interpretability of the model. The priori knowledge means that some initial assumptions or constraints of the model are provided based on domain knowledge, expert experience or existing research results. And in this paper, the GeoStudio software was to generate high quality numerical simulation data based on physical and engineering principles [49]. This data could be used as a priori information to help machine learning models improve their understanding of the relationship between input and output.

**Fig. 14:**
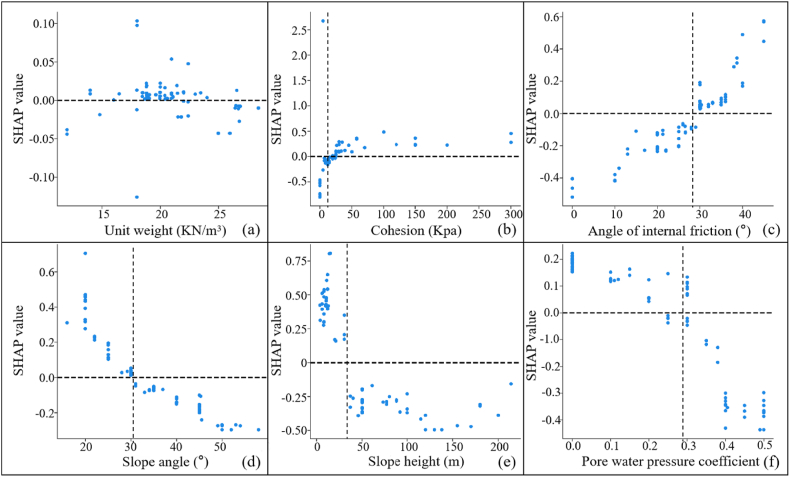
Single factor analysis of 107 groups of GeoStudio data determined by SHAP (a: Unit weight; b: Cohesion; c: Angle of internal friction; d: Slope angle; e: Slope height; f: Pore water pressure coefficient).

Therefore, in this paper, 107 groups of slope data were recalculated by using GeoStudio software, as shown in Appendix C. The new data was brought into the model to get the explanation results as shown in Fig. 14. The results showed that there was a decreasing trend in SHAP values with increasing unit weight, and there was no clear cut-off point. And a large number of SHAP values concentrated between [0, 0.5], implying that the slope stability although will continue to decline, but the decline is slow. The priori data-driven approach was more in line with the simulation results of GeoStudio than the 107 sets of real samples.

In addition, this paper compared the critical values of 107 sets of real samples, the priori data-driven approach and the GeoStudio simulation, as shown in Table 8. The results showed that the priori data-driven approach was more consistent with the results of the numerical simulations than the real samples. Therefore, through the priori data-driven approach, priori knowledge can be fully utilized to improve model interpretability when data are limited or of low quality.

**Table 8:** Comparison of critical values for different outcomes.

Factors	critical value		
	GeoStudio simulation results	SHAP results	
		Real Sample	The priori data-driven
Unit weight		18	
Cohesion	20	30	18
Angle of internal friction	26	24	28
Slope angle	34	42	32
Slope height	28	20	30
Pore water pressure coefficient	0.28	0.37	0.28

In summary, the use of SHAP interpretation results based on real slope samples as decisions or recommendations may be misleading. Since current AI does not have common sense knowledge, dirty data can change the true logic between input and output. The priori data-driven approach effectively overcame the above difficulties. This method provided more reliable and accurate results of explanation compared to real samples, especially when the data were limited or of low quality. Therefore, when using the SHAP algorithm for sensitivity analysis, this paper strongly recommends the use of slope safety coefficients obtained from numerical simulation software calculations rather than real samples. In addition, this paper constructed a fast and accurate slope stability prediction and sensitivity analysis model based on XGBoost and SHAP. Compared with the existing related studies, this paper not only quickly determined the relationship between each factor and slope stability, but also more accurately determined the critical value of factors. However, this paper still has some limitations and shortcomings that need to be focused on in the future.

(1) The uncertainty principle can affect the performance of machine learning in prediction and sensitivity analysis [50]. Although ensemble learning is a common technique for solving uncertainty problems, this paper did not explore data uncertainty in depth. For example, geometric properties and geotechnical characteristics of slopes [51], more general influencing parameters [52], and so on.
(2) In this paper, only 6 each influence parameters were selected as input parameters, but the factors affecting slope stability were much more than these. In the future, more input parameters should be introduced to construct a larger interpretable slope prediction model. For example, rainfall conditions [53,54], land coverage [55], earthquake activity [56], topographic humidity index [57], and so on.
(3) Currently, there was a study suggesting that causal AI may be useful in improving and explaining decisions and recommendations in human organizations, when the outcomes resulting from taking different actions were difficult to predict [58]. For this reason, this method should be discussed in detail in future related studies.
(4) Although this paper correctly performed the slope stability sensitivity analysis by using the priori data-driven approach, misclassification may still occur in real samples. Therefore, future research needs to focus on when real data would show such problems.

## 5 Conclusions

In this paper, a fast and accurate model for slope stability prediction and sensitivity analysis was constructed based on the priori data-driven approach with XGBoost and SHAP. Optuna, SPO, and WOA were used to optimize the key hyperparameters of XGBoost, and then the sensitivity analysis was carried out based on the optimal model and SHAP. The results of the sensitivity analysis of SHAP were validated by using GeoStudio software. The main conclusions obtained were as follows.

(1) The Optuna-XGBoost features faster running speed, better and comparable prediction accuracy, and better resistance to overfitting than PSO-XGBoost and WOA-XGBoost. The average accuracy of the model in 10 fold cross validation in the validation sets was 91.36 % and the AUC, Cohen's Kappa, Accuracy, F-score, TPR and FPR values in the test sets were 0.9, 0.8136, 0.9091, 0.9231, 1 and 0.2, respectively. In addition, its running time was 121.40891 s, an improvement of 89.94 % and 91.34 % compared to PSO-XGBoost and WOA-XGBoost, respectively.
(2) The ranking of importance of factors determined by SHAP was: cohesion > slope height > angle of internal friction > slope angle > pore water pressure coefficient > unit weight. The results were consistent with the simulation results of GeoStudio software. However, the SHAP misjudged the relationship between unit weight and slope stability. GeoStudio simulation results revealed a negative correlation between unit weight, slope angle, slope height and pore water pressure coefficient and slope stability, while a positive correlation between cohesion and angle of internal friction and slope stability. The lack of common-sense knowledge in current AI and the influence of dirty data resulted in the inability of SHAP to effectively explain the true mechanism of slope destabilization, even with the extended dataset.
(3) The priori data-driven approach provided more reliable and accurate explanatory results than real samples, especially when the data were limited or of low quality. The method correctly judged the correlation between factors and slope stability, which was consistent with the simulation results of GeoStudio software. In addition, the results of the priori data-driven approach indicated that the critical values of cohesion, angle of internal friction, slope angle, slope height, and pore water pressure coefficient during slope destabilization respectively were 18 Kpa, 28°, 32°, 30 m, and 0.28. These results were closer to the GeoStudio simulations than the real samples. Therefore, this paper strongly recommends the use of the priori data-driven approach rather than real samples when using SHAP for slope stability sensitivity analysis.

## Data availability statement

The code for this article was implemented by using Python 3.0 in Jupyter Notebook, which was saved in GitHub (https://github.com/linhehe1111/slope-stability/tree/master). The computer hardware information used in this article included AMD Ryzen 5 5600H with Radeon Graphics (CPU), 16 GB (RAM), NVIDIA GeForce RTX 3050 Laptop (GPU). All data were included in the appendix and in the paper.

## CRediT authorship contribution statement

**Hanjie Lin:** Writing – original draft, Software, Conceptualization. **Li Li:** Writing – original draft, Funding acquisition. **Yue Qiang:** Writing – review & editing, Funding acquisition. **Yi Zhang:** Methodology. **Siyu Liang:** Investigation. **Xinlong Xu:** Formal analysis. **Hongjian Li:** Supervision. **Shengchao Hu:** Visualization.

## Declaration of competing interest

The authors declare that they have no known competing financial interests or personal relationships that could have appeared to influence the work reported in this paper.

## Footnotes

Appendix A Supplementary data to this article can be found online at https://doi.org/10.1016/j.heliyon.2024.e35871.

## References

[1] Liu H.L., Ma Y.B., Zhang W.G. (2021). Overview of the application of big data technology in geological disaster prevention and control. Journal of Disaster Prevention and Reduction Engineering (04).

[2] Huang R.Q. (2007). Large scale landslides and their occurrence mechanisms in China since the 20th century. Journal of Rock Mechanics and Engineering.

[3] Cheng Y.M., Lansivaara T., Wei W.B. (2007). Two-dimensional slope stability analysis by limit equilibrium and strength reduction methods. Comput. Geotech..

[4] Cai F., Ugai K. (2004). Numerical analysis of rainfall effects on slope stability. Int. J. GeoMech..

[5] Li L.C., Tang C.A., Zhu W.C., Liang Z.Z. (2009). Numerical analysis of slope stability based on the gravity increase method. Comput. Geotech..

[6] Gao Y.F., Zhang F., Lei G.H., Li D.Y. (2013). An extended limit analysis of three-dimensional slope stability. Geotechnique.

[7] Zhou F.X., Zhu S.W., Liang Y.W., Zhao W.C. (2023). Precise analysis of soil slope stability by variational limit equilibrium method. Journal of Geotechnical Engineering.

[8] Gao Y.F., Wang D., Zhang F. (2015). Research status and prospect of three-dimensional soil slope stability analysis method. Journal of Hohai University.

[9] Liang H., Zhang H. (2010, July). Identification of slope stability based on the contrast of BP neural network and SVM. 2010 3rd International Conference on Computer Science and Information Technology.

[10] Mahdiyar A., Hasanipanah M., Armaghani D.J., Gordan B., Abdullah A., Arab H. (2017). A Monte Carlo technique in safety assessment of slope under seismic condition. Eng. Comput..

[11] Yang T.H., Wang H., Dong X., Liu F.Y., Zhang P.H., D W.W. (2020). Research status, existing problems, and countermeasures of intelligent evaluation of slope stability in open-pit mines. Journal of Coal Science (06).

[12] Khajehzadeh M., Taha M.R., Keawsawasvong S., Mirzaei H., Jebeli M. (2022). An effective artificial intelligence approach for slope stability evaluation. IEEE Access.

[13] Pham K., Kim D., Park S., Choi H. (2021). Ensemble learning-based classification models for slope stability analysis. Catena.

[14] Karir D., Ray A., Bharati A.K., Chaturvedi U., Rai R., Khandelwal M. (2022). Stability prediction of a natural and man-made slope using various machine learning algorithms. Transportation Geotechnics.

[15] Wang Y., Du E., Yang S., Yu L. (2022). Prediction and analysis of slope stability based on IPSO‐svm machine learning model. Geofluids.

[16] Mahmoodzadeh A., Mohammadi M., Farid Hama Ali H., Hashim Ibrahim H., Nariman Abdulhamid S., Nejati H.R. (2022). Prediction of safety factors for slope stability: comparison of machine learning techniques. Nat. Hazards.

[17] Lin S., Zheng H., Han C., Han B., Li W. (2021). Evaluation and prediction of slope stability using machine learning approaches. Front. Struct. Civ. Eng..

[18] Nanehkaran Y.A., Licai Z., Chengyong J., Chen J., Anwar S., Azarafza M., Derakhshani R. (2023). Comparative analysis for slope stability by using machine learning methods. Appl. Sci..

[19] Lundberg S.M., Lee S.I. (2017). A unified approach to interpreting model predictions. Adv. Neural Inf. Process. Syst..

[20] Lundberg S.M., Erion G., Chen H., DeGrave A., Prutkin J.M., Nair B. (2020). From local explanations to global understanding with explainable AI for trees. Nat. Mach. Intell..

[21] Parsa A.B., Movahedi A., Taghipour H., Derrible S., Mohammadian A.K. (2020). Toward safer highways, application of XGBoost and SHAP for real-time accident detection and feature analysis. Accid. Anal. Prev..

[22] Yang C., Chen M., Yuan Q. (2021). The application of XGBoost and SHAP to examining the factors in freight truck-related crashes: an exploratory analysis. Accid. Anal. Prev..

[23] Jas K., Dodagoudar G.R. (2023). Explainable machine learning model for liquefaction potential assessment of soils using XGBoost-SHAP. Soil Dynam. Earthq. Eng..

[24] Zhou X., Wen H., Li Z., Zhang H., Zhang W. (2022). An interpretable model for the susceptibility of rainfall-induced shallow landslides based on SHAP and XGBoost. Geocarto Int..

[25] Aminpour M., Alaie R., Khosravi S., Kardani N., Moridpour S., Nazem M. (2023). Slope stability machine learning predictions on spatially variable random fields with and without factor of safety calculations. Comput. Geotech..

[26] Aminpour M., Alaie R., Kardani N., Moridpour S., Nazem M. (2023). Highly efficient reliability analysis of anisotropic heterogeneous slopes: machine learning-aided Monte Carlo method. Acta Geotechnica.

[27] Lin S., Liang Z., Zhao S., Dong M., Guo H., Zheng H. (2024). A comprehensive evaluation of ensemble machine learning in geotechnical stability analysis and explainability. Int. J. Mech. Mater. Des..

[28] Zou Q., Jiang H., Cui P., Zhou B., Jiang Y., Qin M., Li C. (2021). A new approach to assess landslide susceptibility based on slope failure mechanisms. Catena.

[29] Huang F., Xong H., Chen S., Lv Z., Huang J., Chang Z., Catani F. (2023). Slope stability prediction based on a long short-term memory neural network: comparisons with convolutional neural networks, support vector machines and random forest models. International Journal of Coal Science & Technology.

[30] Wang G., Zhao B., Wu B., Zhang C., Liu W. (2023). Intelligent prediction of slope stability based on visual exploratory data analysis of 77 in situ cases. Int. J. Min. Sci. Technol..

[31] Ahangari Nanehkaran Y., Pusatli T., Chengyong J., Chen J., Cemiloglu A., Azarafza M., Derakhshani R. (2022). Application of machine learning techniques for the estimation of the safety factor in slope stability analysis. Water.

[32] Ng C.W., Crous P.A., Zhang M., Shakeel M. (2022). Static liquefaction mechanisms in loose sand fill slopes. Comput. Geotech..

[33] Li J., Wang F. (2010). Earth and Space 2010: Engineering, Science, Construction, and Operations in Challenging Environments.

[34] Sah N.K., Sheorey P.R., Upadhyaya L.N. (1994). Maximum likelihood estimation of slope stability. Int. J. Rock Mech. Min. Sci. Geomech. Abstracts.

[35] Lu P., Rosenbaum M.S. (2003). Artificial neural networks and grey systems for the prediction of slope stability. Nat. Hazards.

[36] Chen T., Guestrin C. (2016, August). In Proceedings of the 22nd Acm Sigkdd International Conference on Knowledge Discovery and Data Mining.

[37] Shi N., Li Y., Wen L., Zhang Y. (2022). Rapid prediction of landslide dam stability considering the missing data using XGBoost algorithm. Landslides.

[38] Yan J.Q., Shen Z.Y., Lyu J., Liu J.S. (2022). https://link.cnki.net/doi/10.14188/j.1671-8844.2022-03-013.

[39] Devan P., Khare N. (2020). An efficient XGBoost–DNN-based classification model for network intrusion detection system. Neural Comput. Appl..

[40] Akiba T., Sano S., Yanase T., Ohta T., Koyama M. (2019, July). Proceedings of the 25th ACM SIGKDD International Conference on Knowledge Discovery & Data Mining.

[41] Xie W., Nie W., Saffari P. (2021). Landslide hazard assessment based on Bayesian optimization–support vector machine in Nanping City, China. Nat. Hazards.

[42] Qi C., Tang X. (2018). Slope stability prediction using integrated metaheuristic and machine learning approaches: a comparative study. Comput. Ind. Eng..

[43] Hoang N.D., Pham A.D. (2016). Hybrid artificial intelligence approach based on metaheuristic and machine learning for slope stability assessment: a multinational data analysis. Expert Syst. Appl..

[44] Luo Z., Bui X.N., Nguyen H., Moayedi H. (2021). A novel artificial intelligence technique for analyzing slope stability using PSO-CA model. Eng. Comput..

[45] Wei W., Li X., Liu J., Zhou Y., Li L., Zhou J. (2021). Performance evaluation of hybrid WOA-SVR and HHO-SVR models with various kernels to predict factor of safety for circular failure slope. Appl. Sci..

[46] Nasiri H., Homafar A., Chelgani S.C. (2021). Prediction of uniaxial compressive strength and modulus of elasticity for Travertine samples using an explainable artificial intelligence. Results in Geophysical Sciences.

[47] Kostić S., Vasović N., Sunarić D. (2016). Slope stability analysis based on experimental design. Int. J. GeoMech..

[48] Cox Jr L.A. (2021). Information structures for causally explainable decisions. Entropy.

[49] Pei T., Qiu T., Shen C. (2023). Applying knowledge-guided machine learning to slope stability prediction. J. Geotech. Geoenviron. Eng..

[50] Abdar M., Pourpanah F., Hussain S., Rezazadegan D., Liu L., Ghavamzadeh M., Nahavandi S. (2021). A review of uncertainty quantification in deep learning: techniques, applications and challenges. Inf. Fusion.

[51] Nanehkaran Y.A., Licai Z., Chengyong J., Chen J., Anwar S., Azarafza M., Derakhshani R. (2023). Comparative analysis for slope stability by using machine learning methods. Appl. Sci..

[52] Nanehkaran Y.A., Licai Z., Chen J., Azarafza M., Yimin M. (2022). Application of artificial neural networks and geographic information system to provide hazard susceptibility maps for rockfall failures. Environ. Earth Sci..

[53] Yimin M., Yican L., Simon Mwakapesa D., Genglong W., Ahangari Nanehkaran Y., Asim Khan M., Maosheng Z. (2021). Innovative landslide susceptibility mapping portrayed by CA‐AQD and K‐means clustering algorithms. Adv. Civ. Eng..

[54] Mao Y.M., Mwakapesa D.S., Li Y.C., Xu K.B., Nanehkaran Y.A., Zhang M.S. (2022). Assessment of landslide susceptibility using DBSCAN-AHD and LD-EV methods. J. Mt. Sci..

[55] Nanehkaran Y.A., Chen B., Cemiloglu A., Chen J., Anwar S., Azarafza M., Derakhshani R. (2023). Riverside landslide susceptibility overview: leveraging artificial neural networks and machine learning in accordance with the United Nations (UN) sustainable development goals. Water.

[56] Nanehkaran Y.A., Mao Y., Azarafza M., Kockar M.K., Zhu H.H. (2021). Fuzzy-based multiple decision method for landslide susceptibility and hazard assessment: a case study of Tabriz, Iran. Geomechanics and Engineering.

[57] Wang Y., Nanehkaran Y.A. (2024). GIS-based fuzzy logic technique for mapping landslide susceptibility analyzing in a coastal soft rock zone. Nat. Hazards.

[58] Wu C.M., Schulz E., Speekenbrink M., Nelson J.D., Meder B. (2017). Mapping the unknown: the spatially correlated multi-armed bandit. bioRxiv.

